# Torsin ATPases: Harnessing Dynamic Instability for Function

**DOI:** 10.3389/fmolb.2017.00029

**Published:** 2017-05-11

**Authors:** Anna R. Chase, Ethan Laudermilch, Christian Schlieker

**Affiliations:** ^1^Department of Molecular Biophysics and Biochemistry, Yale UniversityNew Haven, CT, USA; ^2^Department of Cell Biology, Yale School of MedicineNew Haven, CT, USA

**Keywords:** AAA+ proteins, TorsinA, dystonic disorders, nuclear membrane, nuclear pore complex, DYT1 dystonia, protein quality control, ubiquitin

## Abstract

Torsins are essential, disease-relevant AAA+ (ATPases associated with various cellular activities) proteins residing in the endoplasmic reticulum and perinuclear space, where they are implicated in a variety of cellular functions. Recently, new structural and functional details about Torsins have emerged that will have a profound influence on unraveling the precise mechanistic details of their yet-unknown mode of action in the cell. While Torsins are phylogenetically related to Clp/HSP100 proteins, they exhibit comparatively weak ATPase activities, which are tightly controlled by virtue of an active site complementation through accessory cofactors. This control mechanism is offset by a TorsinA mutation implicated in the severe movement disorder DYT1 dystonia, suggesting a critical role for the functional Torsin-cofactor interplay *in vivo*. Notably, TorsinA lacks aromatic pore loops that are both conserved and critical for the processive unfolding activity of Clp/HSP100 proteins. Based on these distinctive yet defining features, we discuss how the apparent dynamic nature of the Torsin-cofactor system can inform emerging models and hypotheses for Torsin complex formation and function. Specifically, we propose that the dynamic assembly and disassembly of the Torsin/cofactor system is a critical property that is required for Torsins' functional roles in nuclear trafficking and nuclear pore complex assembly or homeostasis that merit further exploration. Insights obtained from these future studies will be a valuable addition to our understanding of disease etiology of DYT1 dystonia.

## Introduction

Torsin ATPases are essential and broadly conserved AAA+ proteins whose discovery was tied to the characterization of the TorsinA DYT1 mutation found in patients with early-onset torsion dystonia, a highly debilitating hereditary movement disorder (Ozelius et al., 1997). Torsins have recently garnered increasing interest in conjunction with pivotal discoveries about their structure and molecular mechanism of activation, as well as compelling insights into their cellular functions. As the sole AAA+ ATPase found in the endoplasmic reticulum (ER) and nuclear envelope (NE), Torsins were implicated in equally broad and critical functions including lipid synthesis (Grillet et al., 2016), regulation of membrane morphology (Rose et al., 2014), and protein quality control (Chen et al., 2010; Nery et al., 2011) as well as the ER redox sensing (Zhu et al., 2008, 2010; Nery et al., 2011; Zhao et al., 2016).

In addition to these roles in the ER, Torsins fulfill distinct functions at the NE. TorsinA and its cofactor LAP1 are essential for proper assembly of fibroblast nuclear envelope-anchored transmembrane actin-associated nuclear (TAN) lines (Luxton et al., 2011), which are comprised of arrays of linker of nucleoskeleton and cytoskeleton (LINC) complexes associated with retrograde flowing actin. TorsinA modulates the rearward motion of nuclei during centrosome positioning and is implicated in maintaining cell polarity in migrating cells (Saunders et al., 2017). A second intriguing role for Torsins at the nuclear periphery is their involvement in modulating nuclear envelope architecture. Deletions of Torsins in human, mouse, worm, and fly cells lead to the formation of omega-shaped “bleb” compartments within the nuclear envelope (Goodchild et al., 2005; Jokhi et al., 2013; Liang et al., 2014; VanGompel et al., 2015; Laudermilch et al., 2016; Tanabe et al., 2016). These perinuclear blebs have been shown to harbor ubiquitinated proteins (Liang et al., 2014; Laudermilch et al., 2016) as well as nuclear pore complex components (Laudermilch et al., 2016). Thus, a picture is emerging in which Torsins accomplish a variety of tasks both at the NE and the ER, and that at least some of these functions are most critical during early developmental stages in neurons (Tanabe et al., 2016). In addition to these functional insights in the cellular context, the recently solved crystal structures of wild-type and DYT1 dystonia mutant Torsin in complex with its cofactor LULL1 confirmed functionally significant structural features that were previously unappreciated (Demircioglu et al., 2016). Several reviews have summarized the current state of the Torsin field (Rose et al., 2015; Laudermilch and Schlieker, 2016; Cascalho et al., 2017); thus, the purpose of the forgoing is to spotlight current hypotheses surrounding the Torsins' roles at the inner nuclear membrane and their dynamic assembly into an active, functional complex.

## Structural insights into Torsin complexes

**Figure 1:**
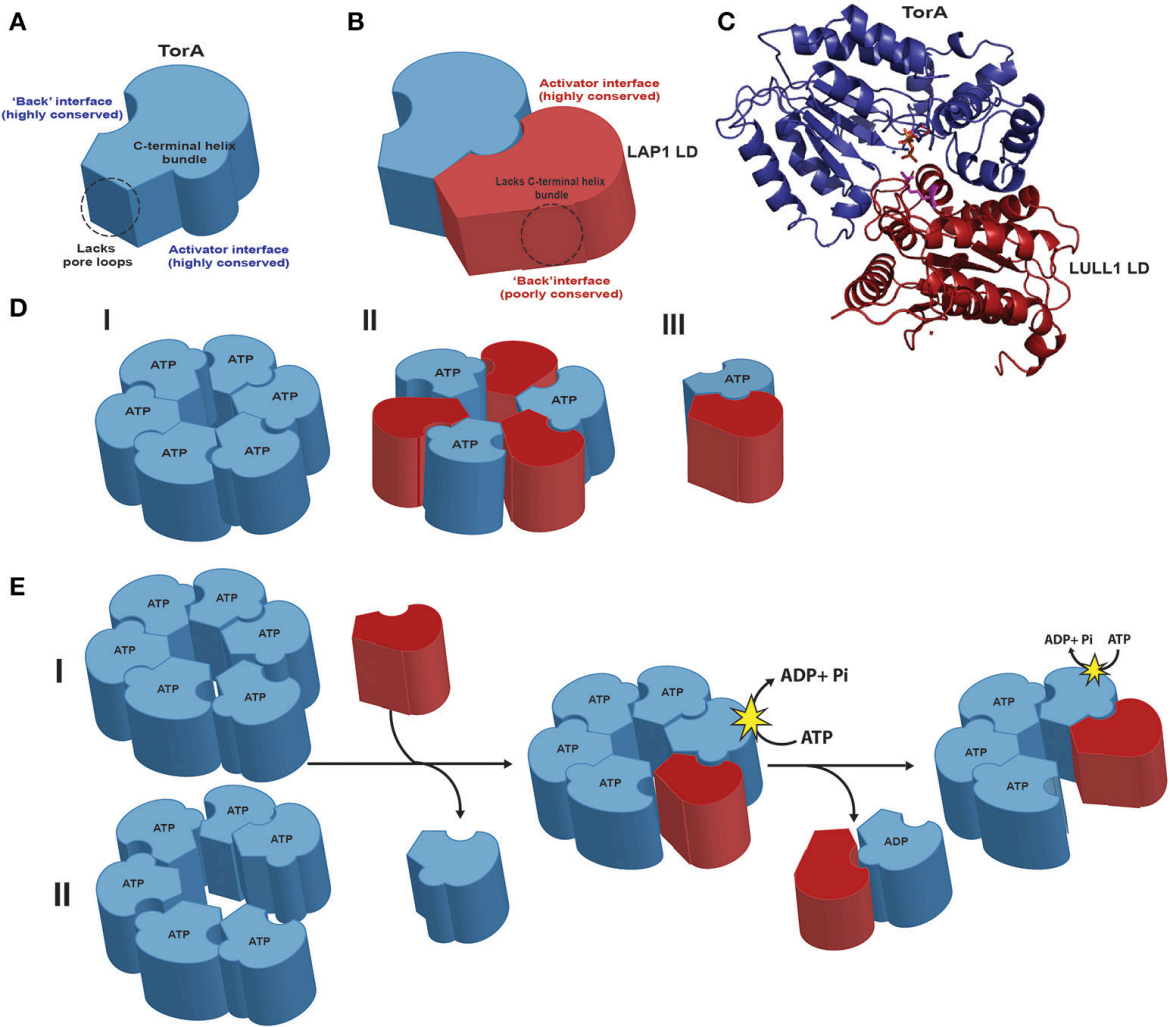
**Structural features of TorsinA and its dynamic complexes with cofactors (A)**. TorsinA (blue) exhibits high levels of conservation both on the activator and “back” interface. Torsins have a C-terminal helix bundle that serves to form intra-protomer contacts in related AAA+ proteins but lack the aromatic pore loops that usually serve to thread substrates through the central pore. The membrane-associated N-terminal hydrophobic domain was omitted for clarity. **(B)** The cofactor LAP1 (maroon) luminal domain, which adopts a AAA+ fold, lacks the critical four-helix bundle and exhibits a low level of conservation on its “back” interface opposite the more conserved activator binding face. **(C)** Cartoon representation of the TorsinA-LULL1 crystal structure (PDB code 5J1S; the nanobody used for crystallization was omitted for clarity). Note that the luminal domains of LAP1 and LULL1 are 60% identical. The cofactor/Torsin complex features a tightly apposed interface in the presence of ATP (orange), with the cofactor supplying a catalytic arginine finger (magenta) that reaches into the nucleotide binding site of Torsin to activate its ATPase activity. **(D)** Three different models exist for the active assembly of Torsins: (I) a homo-oligomeric (likely hexameric) ring; (II) a trimer of heterodimers; (III) a Torsin-LAP1 heterodimer. **(E)** Predicted model of active Torsin complex formation with its cofactors. Torsin forms homo-oligomeric complexes in the presence of nucleotide that could adopt either a planar (I) or a stacked spiral (II) conformation. Cofactor binding to the Torsin active site destabilizes the Torsin ring. Torsin-Torsin rings are eventually dismantled because the cofactors lack the necessary four-helix bundle and conserved residues to form stable closed ring structures. The Torsin-cofactor complex is also transient and dynamic: ATP hydrolysis generates ADP-bound Torsin, destabilizing both the Torsin-Torsin and the Torsin-cofactor interaction. Note that the transmembrane domain of LAP1 was omitted for clarity.

Though homology to other AAA+ proteins suggested that Torsins were capable of ATP hydrolysis-driven mechanical work from the very beginning, the question of whether they were active ATPases or degenerate AAA+ scaffolds was unresolved until Torsins were functionally reconstituted *in vitro* (Zhao et al., 2013). TorsinA, -B, and -3A have ATPase activity in the presence of ATP and the luminal domain of the ER-resident protein LULL1 while TorsinA and -B alone are activated by the luminal domain of LAP1, which resides in the NE (Foisner and Gerace, 1993; Goodchild and Dauer, 2005; Zhao et al., 2013). The DYT1 dystonia mutant of TorsinA is refractory to the activation by these cofactors, thus presenting one line of evidence for a loss-of-function mechanism in early-onset torsion dystonia (Zhao et al., 2013). These cofactors have degenerate AAA+ scaffolds lacking the motifs needed for ATP binding, and they activate Torsin ATPase activity by complementing the Torsin active site with an arginine finger residue that is absent in Torsins (Brown et al., 2014; Sosa et al., 2014) (Figures 1A–C).

The structure of the TorsinA-LULL1 heterodimer unambiguously confirmed the critical role of a catalytic arginine (Demircioglu et al., 2016). This arginine is positioned to stabilize the negative charge of the transition state, thus lowering the free energy of the nucleotide hydrolysis reaction (Scheffzek et al., 1998). As suggested by biochemical studies (Brown et al., 2014; Rose et al., 2014) the TorsinA-LULL1 crystal structure confirmed the critical role of Torsin's C-terminal helix region for forming interactions with LULL1 (Demircioglu et al., 2016) (Figure 1C). It is now apparent that the deletion of E303 in the DYT1 dystonia mutant TorsinA perturbs a critical helix at the cofactor interface (Demircioglu et al., 2016), providing an atomic-level rationale for the observation of reduced cross-linking of the conserved C-terminal TorsinA aromatic residues with the cofactor in the TorsinA disease variant (Brown et al., 2014), and the resulting failure to trigger ATP hydrolysis (Zhao et al., 2013) (for additional details on disease implications, see Rose et al., 2015; Cascalho et al., 2017).

The complementation mechanism for ATPase activation and the presence of a degenerated AAA+ fold is unusual but not unprecedented. The bacterial clamp loader has an inactive δ′ subunit that activates the adjacent γ ATP-binding AAA+ subunit (Hedglin et al., 2013; Kelch, 2016). Torsins and their cofactors stand out for the fact that they have different modes of staying anchored in their cellular environment: TorsinA and -B have an N-terminal signal sequence followed by a hydrophobic domain while Torsin2A and -3A do not have a hydrophobic domain, and LULL1 and LAP1 are type-II transmembrane proteins. LULL1 is localized throughout the ER (Goodchild and Dauer, 2005), while the nuclear domain of LAP1 binds to the nuclear lamina and therefore resides in the inner nuclear membrane (Foisner and Gerace, 1993). From an evolutionary standpoint, the added complexity of such a distinctive multi-component ATPase system likely evolved out of the need to create more diverse roles at precise cellular loci, especially in higher organisms. Dependence on the cofactors for at least some of their functions likely allows cells to leverage the common Torsin scaffold to perform more varied functions in targeted locations and potentially relay signals from or to the nucleus and cytoplasm as well.

Though the stoichiometry of the Torsin/cofactor complex under equilibrium conditions remain to be established, recent data point to a dynamic assembly. Three distinct models exist: (a) an alternating, symmetric Torsin/cofactor ring assembly; (b) homo-oligomeric Torsin rings; and (c) a Torsin/cofactor dimer (Figure 1D). Though low-resolution structural (Sosa et al., 2014) data and crosslinking data (Brown et al., 2014) are consistent with the formation of an alternating assembly into a closed ring structure, the major limitation of several approaches aimed at a determination of the (hetero)oligomeric state is that they were mostly carried out with hydrolysis-deficient “trap” variants of TorsinA. These variants are refractory to cofactor-induced hydrolysis (Zhao et al., 2013) and bind the cofactor tightly (Naismith et al., 2009; Zhu et al., 2010; Zhao et al., 2013), a situation that is certainly not representative of the dynamic equilibrium in a cell. The rationale for the second model with Torsin-Torsin homo-oligomers is based on data showing that Torsin assembles into hexameric structures on its own in blue native PAGE experiments, and that ATP is often required to allow oligomerization in AAA+ ATPases (Hanson and Whiteheart, 2005; Vander Heyden et al., 2009; Jungwirth et al., 2010).

Given that previous studies of Torsins were conducted primarily with “trap” variants that resulted in more static models, we propose a more dynamic model. This model is most strongly supported by the following evidence: only Torsins, but not LAP1 and LULL1, possess the C-terminal helix bundle that is essential for intra-protomer ring-forming contacts (Figures 1A–C) among the Clp/Hsp100 AAA+ proteins (Mogk et al., 2003); the high level of conservation observed in Torsin residues on the “back” interface opposite the cofactor binding face (Figures 1A,B) (Demircioglu et al., 2016) suggesting that these residues participate in homotypic Torsin intra-protomer contacts; and the observation of higher-order Torsin oligomers (cf. Figure 1D) via blue native PAGE (Vander Heyden et al., 2009; Jungwirth et al., 2010; Goodchild et al., 2015). Given the cofactors' lack of a four-helix bundle and the low level of “back” interface conservation on either cofactor (Demircioglu et al., 2016), and the fact that Torsin oligomerization itself is ATP-dependent, it is conceivable that activation of ATP hydrolysis by the bound cofactors would effectively disrupt homotypic intra-ring contacts, as proposed previously (Rose et al., 2015; Demircioglu et al., 2016).

One important point of discussion in the context of this model is how the cofactor luminal domains, which would effectively compete with other Torsin subunits in the ring for a nearly identical interface would manage to initially pervade the ring, gaining access to an ATP-bound Torsin subunit. One possibility (Figure 1E–II) is that Torsin oligomers adopt a split lock washer or spiral conformation, similar to NSF (Zhao et al., 2015), in which parts of the nucleotide binding face of Torsin would be rendered accessible to the cofactor. The flexibility of the unstructured region after the hydrophobic domain but before the AAA+ domain (residues 44-57) could impart additional degrees of translational freedom (a ~49 Å radius of flexibility, based on Cα-Cα distance) to Torsin subunits, thus also allowing the membrane-anchored cofactors to access the nucleotide binding site, which is about 30 Å from the membrane-anchored N-terminus. Considering that ATP binding is broadly required for oligomerization in AAA+ ATPases, hydrolysis and transition to the ADP-bound state would shift the equilibrium to free Torsin and cofactor subunits (Figure 1E I-II). Adding to the complexity of the system is the fact that LULL1 has been shown to form higher-order structures (Goodchild et al., 2015), thus creating an equilibrium reaction between Torsin-engaged, free, and homo-oligomeric or otherwise engaged cofactors. Furthermore, it is possible that the cofactors are themselves regulated by an additional layer of control: for example via posttranslational modifications, through dynamic interactions with other proteins on either side of the membrane, or even within the lipid bilayer. In either case, the known properties of the Torsin-cofactor complex are not consistent with a static assembly.

Unlike the Clp/Hsp100 proteins which Torsins are most phylogenetically similar to, the Torsin structure (Demircioglu et al., 2016) further established that Torsins lack the central hydrophobic pore loops that are used to drive substrate translocation through the central channel of other related hexameric AAA+ proteins (Olivares et al., 2016). Combined with the extremely slow ATPase activity (0.006 nucleotides/s), relative to its AAA+ counterparts which can hydrolyze >1.3 nucleotides/s (Martin et al., 2008), these observations render it improbable that Torsin acts in a processive manner to translocate substrates through the inner cavity of the Torsin ring (Zhao et al., 2013; Rose et al., 2015). Instead, Torsins likely interact with substrates with a more transient mechanism such as that of a holder chaperone that quickly binds and releases its substrates, either by lateral diffusion into the axial pore or by binding substrates at the periphery of its assembly. Determining the three dimensional structure of higher-order Torsin assemblies using e.g., cryo-electron microscopy might provide important insights in the future. Though characterizing the precise mechanisms of how ATP hydrolysis translates to work exerted on substrates remains challenging even for well-characterized AAA+ proteins, recent studies on NSF, the yeast chaperone Hsp104, and mitochondrial Pex1/Pex6 by cryo-EM have revealed that progression through multiple asymmetric states in stacked spirals, open lock-washers, or more planar assemblies are key drivers for performing work during successive ATP hydrolysis events (Blok et al., 2015; Zhao et al., 2015; Yokom et al., 2016). Given the Torsins assembly's dynamic nature, predicted non-processive action, and similarity to clamp loaders, it is probable that the presence of asymmetric states will also play a role in its activation mechanism and should be accounted for in data analysis and interpretation. Asymmetric hydrolysis events could, for example, couple various asymmetric states to the insertion of the Torsins' own hydrophobic domains or interaction with the transmembrane cofactors, which could in turn modulate membrane curvature and remodeling or substrate interactions. It will be important to examine these states both in the presence and absence of cofactors and, once they have been identified, the Torsin substrates that have eluded the field thus far.

How can we begin to form a mechanistic explanation for the Torsins' exquisite spatiotemporal control during phases of neuronal development while also accounting for their redundancy (Laudermilch et al., 2016; Tanabe et al., 2016)? One likely scenario, is the formation of an anti-parallel gradient by the cofactors LULL1 in the ER and LAP1 at the nuclear envelope that dictate when and where Torsins are activated by cofactors to perform their function (Rose et al., 2015). LULL1 could activate Torsin's chaperone function in the ER, perhaps in response to a flux in redox potential or cofactor density in this compartment. The membrane association of TorsinA is controlled by cleavage of a scissile bond that removes the N-terminal hydrophobic domain during B cell differentiation (Zhao et al., 2016), suggesting an additional layer of control that could modulate substrate specificity, for example from membrane-associated to soluble ER-luminal species, during ER expansion. TorsinA species with a mass identical to this cleavage product have been observed in organ homogenates (Goodchild et al., 2005; Jungwirth et al., 2010).

## A novel role for Torsins in nuclear pore biogenesis or homeostasis

**Figure 2:**
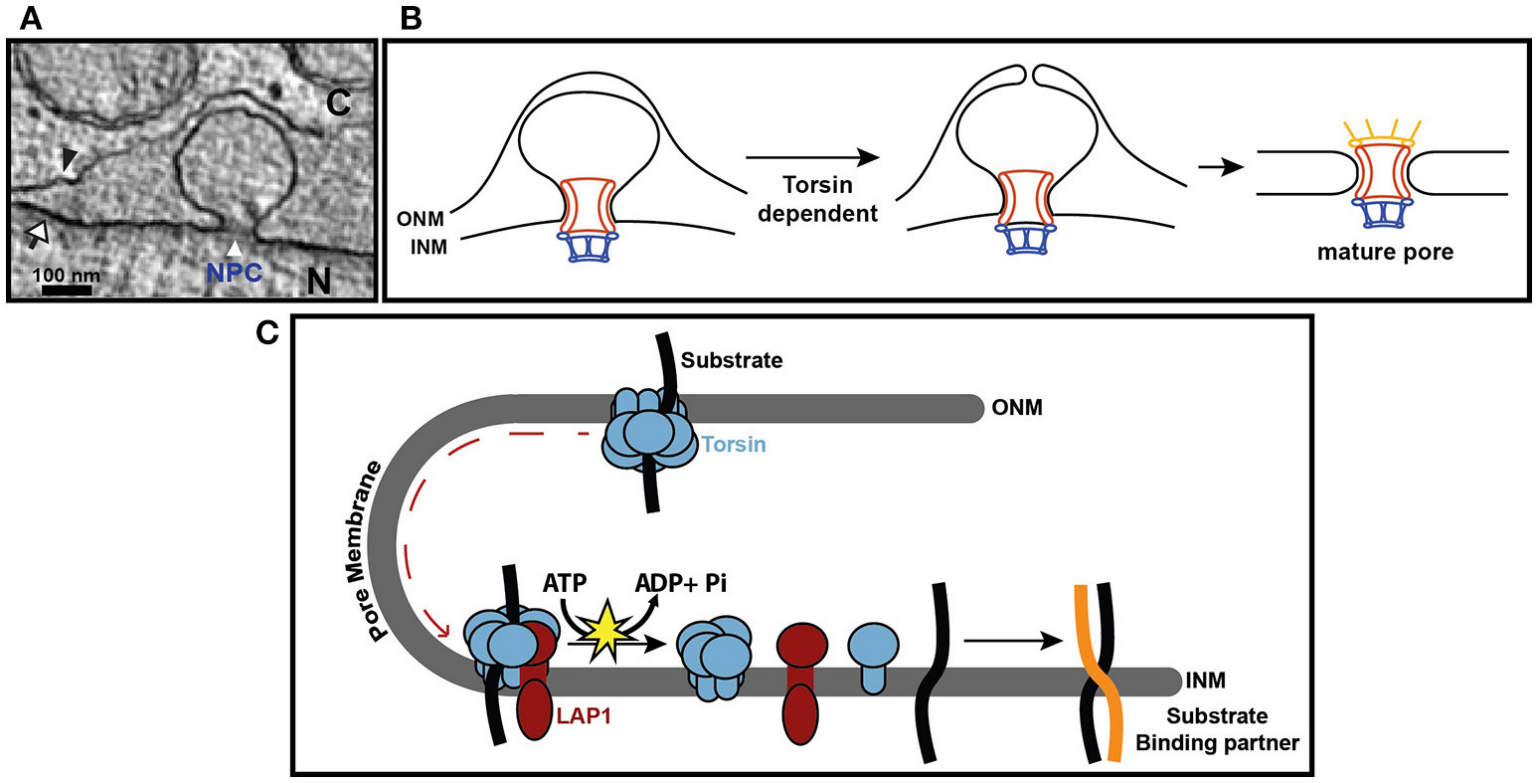
**Torsin function at the nuclear envelope**. **(A)** EM cross section of nuclear envelope blebbing observed in Torsin-deficient HeLa cells. N, nucleus; C, cytoplasm; black arrowhead, ONM; white arrow, INM; white arrowhead, electron density at the base of the blebs containing nucleoporins. **(B)** Model depicting how blebs could arise from stalled NPC assembly. In this model, Torsin would function at a step prior to or at membrane fusion. **(C)** Hypothetical model for Torsins as a trafficking chaperones that deliver proteins to the inner nuclear membrane. INM-resident proteins are sequestered by Torsins during de novo synthesis in the ER or the contiguous ONM, preventing their premature assembly into protein-protein complexes that would compromise or prevent their trafficking through the pore membrane. Upon arrival at the INM, the high local concentration of LAP1 would trigger ATP hydrolysis in Torsins, leading to the disassembly of the Torsin ring and substrate release. Released substrates can then engage in protein-protein complex formation at the INM.

The hallmark phenotype seen upon Torsin manipulation or deletion is the “blebbing” or herniation of the inner nuclear membrane into the perinuclear space (Figure 2A; Goodchild et al., 2005; Jokhi et al., 2013; Liang et al., 2014; Pappas et al., 2015; VanGompel et al., 2015; Laudermilch et al., 2016; Tanabe et al., 2016). This phenotype has been observed in neural tissues of knockout mouse models of TorsinA (Goodchild and Dauer, 2005) and in HeLa cells with combined knockouts of multiple Torsins (Laudermilch and Schlieker, 2016). Similar herniations have also been observed after manipulation of the respective Torsin variants in *Drosophila melanogaster* and *Caenorhabditis elegans* (Jokhi et al., 2013; VanGompel et al., 2015), suggesting that Torsin function at the nuclear envelope is conserved.

One formidable challenge to deciphering Torsin function has been the remarkable redundancy between the four Torsin proteins encoded in mammalian genomes. In TorsinA knockout mice, blebbing is observed strictly in neural tissue (Goodchild et al., 2005), where TorsinA is highly expressed (Jungwirth et al., 2010). However, in fibroblasts from TorsinA knockout mice, additionally depleting TorsinB is sufficient to induce blebbing (Kim et al., 2010). In TorsinA knockout mice, blebbing is restricted to a specific developmental window, and the resolution of the blebs in later stages is dependent on increasing expression levels of TorsinB (Tanabe et al., 2016). Finally, deletion of TorsinA or TorsinB individually in HeLa cells shows little perturbation to normal nuclear envelope architecture, but deleting all four Torsins results in robust blebbing (Laudermilch et al., 2016).

While the precise composition of the blebs and Torsins' role in their formation is still being determined, several recent findings linked Torsins to nucleoporins (nups) (VanGompel et al., 2015; Laudermilch et al., 2016). In *C. elegans*, Torsin manipulation resulted in nup mislocalization and altered nuclear import kinetics (VanGompel et al., 2015). In Torsin-deficient HeLa cells, a subset of nups localize specifically to the base or “neck” of the blebs at the inner nuclear membrane (Laudermilch et al., 2016) (Figure 2A). Collectively, these observations suggest that Torsin plays a role in nuclear pore complex (NPC) biogenesis or homeostasis. The NPC is a massive structure found in the nuclear envelope through which nucleocytoplasmic transport occurs (Field et al., 2014; Knockenhauer and Schwartz, 2016; Kosinski et al., 2016; Lin et al., 2016). While the precise mechanism of NPC assembly is still actively investigated, there are two distinct assembly pathways: one occurs post-mitotically while the nuclear envelope reforms and the other occurs during interphase (Doucet et al., 2010). Interphase assembly begins from the INM and proceeds outward toward the ONM. After several subcomplexes have assembled, the inner and outer nuclear membranes fuse together, and at least some components of the cytoplasmic region are added to the NPC after this fusion event (Otsuka et al., 2016).

Here we propose two models for a functional link between Torsins and nups. Importantly, the shape and dimensions of the blebs are highly similar to normal interphase NPC assembly intermediates (Laudermilch et al., 2016; Otsuka et al., 2016). Thus, the blebs could represent frozen NPC assembly intermediates that require the action of Torsins for their completion. These intermediates would be frozen at a step *prior* to the fusion of the inner and outer nuclear membranes (Figure 2B). Thus, cytoplasmic nups would be expected to be absent from the base of the blebs in this model, while other subcomplexes would be present. Therefore, it will be critical to perform a detailed compositional analysis of the blebs. A diagnostic absence of cytoplasmic nups would support the idea of a frozen assembly intermediate. That Torsin-deficient cells remain viable albeit exhibiting slower growth (Laudermilch et al., 2016) could be attributed to the contribution of unperturbed NPC assembly proceeding through the Torsin-independent postmitotic insertion pathway.

Alternatively, the blebs could result from sealing of nascent NPCs by endosomal sorting complexes required for transport (ESCRT) components, analogous to a process that has recently been described in yeast in which ESCRT proteins and the AAA+ ATPase Vps4 participate in a pathway that surveils NPCs (Webster et al., 2014; Webster and Lusk, 2016).

We envision two general mechanistic models to explain why blebs form in the absence of Torsin. In the first model, Torsin would act directly in NPC biogenesis. For example, Torsin might participate in the fusion of the inner and outer nuclear membranes during NPC assembly, probably in complex with other proteins. In the second model, Torsin would act upstream of NPC biogenesis or surveillance. Specifically, Torsins could act as trafficking chaperones by binding newly synthesized proteins in the endoplasmic reticulum and delivering them to sites of NPC assembly in the nuclear envelope (Figure 2C). Torsin could traffic transmembrane nups, or it could deliver proteins that are essential for NPC assembly or surveillance. One reason for invoking such a function is the presence of a 60 kDa transport limit for the nuclear domains of transmembrane proteins residing in the INM (Ungricht et al., 2015). NE proteins assembling into higher-order oligomeric structures must be held competent for trafficking through the pore membrane in a monomeric state to bypass the 60 kDa size limitation imposed by the NPC. For example, trimeric Sun proteins (Sosa et al., 2012) at INM harbor sizable nuclear domains (~34 kDa for Sun1). Trafficking through the pore membrane in a trimeric state would be difficult to reconcile with this 60 kDa size limit. Our specific proposal here is that Torsins could stabilize the monomeric form by association with the luminal domains of NE proteins, while the nuclear domains of NE proteins will ensure INM targeting. Upon arrival at the INM, substrates will be released from Torsins due to the high local concentration of the Torsin activator LAP1 at the INM resulting in disassembly of the Torsin ring and allowing the released substrate to engage in complex formation (Figure 2C). While hypothetical, this model would be consistent with the observation that a hydrolysis-deficient trap variant of TorsinA accumulates in the NE (Goodchild and Dauer, 2004; Naismith et al., 2004), which can be attributed to a failure of LAP1 to catalyze the release of Torsin from its NE-targeted clients.

Our model could also explain the accumulation of K48-ubiquitylated proteins in the nuclear periphery in Torsin deficient cells (Laudermilch et al., 2016). Given that the INM of mammalian cells was recently shown to be competent for the degradation of membrane proteins (Tsai et al., 2016), it will be critical to determine if the half life of otherwise stable NPC/INM proteins (Doucet et al., 2010; Toyama et al., 2013) is compromised in Torsin-deficient cells due to the absence of normally stabilizing interactions that are perturbed due to trafficking defects, and to discern a (mis)localization of INM proteins to the ONM vs. INM upon Torsin manipulation.

In conclusion, we have now reached a stage in our understanding of Torsin biology that is sufficient to begin formulating more precise hypotheses about their mechanism and their functions that can be tested by definitive experiments. The likelihood that further genetic experiments within a cellular context will yield the holy grail of the Torsin field—the elusive substrates that trigger the changes affected by Torsins in the ER and at the nuclear envelope—is more probable than ever. Merging these functional details with a structural understanding of the Torsins' action will provide the necessary basis for developing targeted DYT1 dystonia therapies.

## Author contributions

All authors listed, have made substantial, direct, and intellectual contribution to the work, and approved it for publication.

### Conflict of interest statement

The authors declare that the research was conducted in the absence of any commercial or financial relationships that could be construed as a potential conflict of interest.
